# Genetic-epigenetic interactions (meQTLs) in orofacial clefts etiology

**DOI:** 10.1007/s00438-026-02474-4

**Published:** 2026-06-23

**Authors:** A. L. Petrin, L. A. Machado-Paula, H. L. Keen, L. Hovey, B. Doolittle, L. Dunlay, W. Awotoye, X. J. Xie, E. Zeng, A. Butali, M. L. Marazita, J. C. Murray, L. M. Moreno-Uribe

**Affiliations:** 1 College of Dentistry and Dental Clinics, University of Iowa , Iowa City, IA 52242 USA; 2 University of Iowa Carver College of Medicine, Iowa City, IA USA; 3 Tufts University School of Dental Medicine, Boston, MA USA; 4 University of North Carolina Adams School of Dentistry, Chapel Hill, NC USA; 5 Florida State University College of Medicine, Talahasse, FL USA; 6 School of Dental Medicine, University of Pittsburgh, Pittsburgh, PA USA

**Keywords:** MeQTLs, Cleft lip and palate, Discordant siblings, DNA methylation, Epigenetics, Orofacial clefts

## Abstract

Understanding how genetic variants influence disease risk through molecular mechanisms remains a central challenge in complex disease genetics. Nonsyndromic orofacial clefts (OFCs) exemplify this challenge, with most risk loci residing in non-coding regions. We hypothesized that common genetic variants influence OFC risk by modulating DNA methylation at regulatory elements through methylation quantitative trait loci (meQTLs). We analyzed 10 OFC-associated SNPs against genome-wide DNA methylation profiles in 409 cases and 456 controls, identifying 23 potential meQTLs. Findings were validated using 358 cleft-discordant sibling pairs with MethyLight assays. We performed formal mediation analysis, genotype-tissue interaction and cross-referenced with the mQTL Database to assess developmental timing. Nine meQTLs were validated, including rs987525 (8q24)–cg16561172 (*MYC*) (*P* = 9.6 × 10⁻⁶), which mapped to a mesendoderm-active enhancer upstream of *MYC*. Genotype × tissue interaction confirmed tissue-specificity (*P* = 1.00 × 10^− 3^), with stronger effects in oral-derived tissue (saliva). Additional validated SNP-CpG associations involved *MAFB*–*PLCG1*, *NOG*–*PPM1E*, *FOXE1*–*FRZB*, and *SPRY2*–*LGR4*. While effect sizes correlated between tissues (*r* = 0.81), formal mediation analysis indicated individual CpG sites do not fully mediate SNP-phenotype relationships, suggesting coordinated epigenetic mechanisms. Most associations showed peak effects during childhood, while 8q24 showed unique adult-specific patterns. We identified genetic variants influencing methylation at craniofacial regulatory elements, and provided a mechanistic link for a major risk locus, 8q24, with tissue-specific effects in saliva. While individual CpG sites did not fully mediate the genetic risk, our findings identified specific regulatory regions where coordinated epigenetic changes may contribute to OFC susceptibility.

## Introduction

Genome-wide association studies have identified genetic variants associated with hundreds of human diseases, yet translating these statistical associations into biological mechanisms remains a fundamental challenge in genetics. Nonsyndromic orofacial clefts (OFCs) exemplify this gap: despite affecting approximately 1 in 700 live births worldwide and representing the most common craniofacial birth defects in humans (Rahimov, Jugessur et al. 2012), the functional mechanisms underlying most identified risk variants remain unknown. Over 60 genetic loci have been associated with OFCs, but the majority reside in non-coding genomic regions, raising the critical question of how these variants influence disease risk. DNA methylation—an epigenetic modification that regulates gene expression throughout development—offers a mechanistic bridge between genetic variation and phenotypic outcomes. By systematically characterizing methylation quantitative trait loci (meQTLs) at established OFC risk variants, this study aimed to identify the molecular pathways through which genetic variation contributes to cleft susceptibility.

The clinical impact of OFCs extends beyond the immediate structural defect, with affected individuals facing challenges in feeding, speech development, hearing, dental health, and psychosocial well-being throughout their lives (Fraser 1955; Marazita 2012). While family history is observed in approximately 23% of cases, indicating substantial genetic contribution, monozygotic twins show only 50% concordance rates (Grosen, Bille et al. 2010). This incomplete penetrance, combined with the observation that concordant twins often exhibit different phenotypic severity, suggests that factors beyond primary genetic risk variants—potentially including epigenetic modifications—contribute to disease manifestation and phenotypic variability.

Together, GWAS (Birnbaum, Ludwig et al. 2009; Grant, Wang et al. 2009; Beaty, Murray et al. 2010; Mangold, Ludwig et al. 2010; Camargo, Rivera et al. 2012; Sun, Huang et al. 2015; Wolf, Brand et al. 2015; Leslie, Liu et al. 2016), GWAS meta-analyses (Ludwig, Mangold et al. 2012; Beaty, Taub et al. 2013), linkage (Moreno, Mansilla et al. 2009), and replication studies have identified over 60 risk loci for OFCs; however, these account for a minority of estimated heritability, and many reside in non-coding regions with unclear functional relevance. This challenge in translating statistical associations into biological mechanisms necessitates expansion beyond single-omics analyses.

Recent advances in epigenomics have revealed DNA methylation as a critical mechanism through which genetic variants can influence gene expression and disease susceptibility. DNA methylation, involving the covalent addition of methyl groups to cytosine residues in CpG dinucleotides, serves as a key epigenetic modification regulating gene expression throughout development and adult life. Importantly, DNA methylation patterns can be influenced by both genetic variation and environmental factors, making them attractive candidates for explaining gene-environment interactions in complex diseases(Bourc’his, Xu et al. 2001; Song, Rechkoblit et al. 2011; van Eijk, de Jong et al. 2012).

Methylation quantitative trait loci (meQTLs)—genetic variants that significantly influence DNA methylation levels at specific CpG sites—provide a mechanistic framework for understanding how non-coding variants affect gene regulation. Large-scale studies have demonstrated that genetic effects on DNA methylation are widespread, with recent analyses showing that up to 45% of CpG sites are influenced by genetic variants (Villicaña and Bell 2021; Villicaña, Castillo-Fernandez et al. 2023). For OFCs, where many risk variants are located in non-coding regions near developmental genes, meQTL analysis offers an approach to identify functional mechanisms underlying these associations.

Emerging evidence supports that differential DNA methylation may alter risk for different cleft types and modify OFCs penetrance (Alvizi, Ke et al. 2017). Our recent work showed that differential methylation contributes to phenotypic variability in patients with Van der Woude syndrome, including monozygotic twins with discordant phenotypes despite carrying identical causal mutations in the *IRF6* gene (Petrin, Zeng et al. 2023; Seaberg, Awotoye et al. 2024). Additional studies indicate that epigenetic factors such as DNA methylation are associated with OFCs risk (Joubert, Felix et al. 2016; Alvizi, Ke et al. 2017; Sharp, Ho et al. 2017; Shu, Shu et al. 2018; Gonseth, Shaw et al. 2019; Xu, Lie et al. 2019; Young, Slifer et al. 2021; Alvizi, Brito et al. 2022b; Charoenvicha, Sirimaharaj et al. 2022; Zhang, Zhang et al. 2023) with differentially methylated regions tending to cluster around gene pathways previously linked to palatogenesis. However, systematic characterization of meQTLs specifically in the context of OFC-associated genetic variants has not been performed, representing a significant knowledge gap in understanding functional mechanisms underlying genetic risk.

Therefore, this study combines known genetic risk factors with epigenetic data to identify and validate meQTLs in OFC etiology. We employed a discovery cohort of unrelated cases and controls followed by validation in cleft-discordant sibling pairs—a design that controls for genetic background and shared environmental factors while maintaining power to detect disease-relevant methylation differences (Kim, Kwak et al. 2017). Our objectives were to (1) identify meQTL associations between established OFC risk variants and CpG sites genome-wide using a cohort of cases and controls, (2) validate associations in an independent cohort using a complementary methylation measurement approach, and (3) characterize the functional and developmental context of validated meQTLs.

## Methods

### Study design and samples

Our study employed two cohorts to identify and validate meQTLs associated with orofacial clefts. The discovery cohort (Cohort 1) consisted of unrelated cases (individuals with cleft lip with or without cleft palate) and controls with genome-wide methylation data, while the validation cohort (Cohort 2) comprised of same-sex cleft-discordant sibling pairs to control for genetic background and shared environmental factors. All samples have been previously collected as part of different studies, all approved by their respective IRBs and by the University of Iowa IRB. Informed consent was obtained for all individual participants included in the study.

### Cohort 1 (Discovery)

We used genetic and epigenetic data from a subset of unrelated cases with cleft lip with or without cleft palate (*N* = 409) and unaffected controls (*N* = 456) (Moreno Uribe, Fomina et al. 2017; Romanowska, Haaland et al. 2020). We obtained genotype data for 10 cleft-associated SNPs for all individuals, along with genome-wide DNA methylation data obtained with the Illumina 450 K array. All cohort 1 DNA was extracted from blood. The 10 SNPs included were selected for their role as top loci for OFCs based on previous GWAS and candidate gene studies.

### Cohort 2 (Validation)

 The validation cohort consisted of 358 pairs of same-sex siblings discordant for cleft lip with or without cleft palate with DNA from blood (*n* = 164 pairs) and DNA from saliva (*n* = 194 pairs) (Table 1). Cohort 2 showed balanced sex distribution (51% male, 49% female) and representation across multiple populations, with Asian populations comprising the largest group (33.2%), followed by South American (26.2%), North American (20%), African (8%), European (7%), and Central American (5%) populations. DNA samples were obtained from blood in 46% of cases and saliva in 54% of cases. Among affected individuals, cleft lip and palate (CLP) represented the most common phenotype (71%), followed by isolated cleft lip (CL, 22%) and isolated cleft palate (CPO, 7%).

Samples from both cohorts were obtained as part of previous studies following approval by the University of Iowa and local Institutional Review Boards (IRBs). Informed consent was provided by patients, parents, or guardians prior to sample collection and clinical information acquisition.

**Table 1:** Distribution of samples from cohort 2

Characteristic	Total samples (*N*)/Sib pairs (*N*)	%
*Total Samples*	716/358	100%
*Gender*		
Male	364/182	51%
Female	352/176	49%
*Sample Source*		
Blood	328/164	46%
Saliva	388/194	54%
*Cleft Type*		
CL	80	22%
CLP	254	71%
CPO	24	7%
*Population*		
Asia	238	33.2%
South America	188	26.2%
North America	146	20.0%
Africa	60	8.0%
Europe	48	7.0%
Central America	36	5.0%

*N* number of samples, *CL *cleft lip, *CLP* cleft lip and palate, *CPO *cleft palate only

### Genotyping

We selected 10 SNPs representing well-established OFC risk loci based on their identification as genome-wide significant associations in previous GWAS and meta-analyses (Romanowska, Haaland et al. 2020) (Moreno Uribe, Fomina et al. 2017) (Table 2). These SNPs were chosen to represent the strongest and most validated genetic associations with OFCs, spanning different chromosomes and putative functional mechanisms.

For cohort 1, we obtained genetic data for the selected 10 SNPs for all individuals from a previous study. Detailed information regarding data collection, quality control, and preprocessing is available in the appendix of the cited work (Romanowska, Haaland et al. 2020) (Moreno Uribe, Fomina et al. 2017). For cohort 2, we genotyped the 10 SNPs in the discordant sibling pairs using Taqman assays on a Fluidigm nanofluidic platform (Fluidigm Corp., South San Francisco, CA, USA). Genotype calling was performed using Fluidigm SNP genotyping software version 4.1.2 with default settings. Quality control included setting the confidence threshold to 65% for the genotype calling algorithm followed by visual inspection of all genotyping plots.

### Bisulfite conversion and DNA methylation profiling

All samples were bisulfite converted prior to measuring DNA methylation levels. Briefly, DNA quality was assessed QubitTM dsDNA High Sensitivity Range Assay Kit (Thermo Fisher Scientific) and 1.5% agarose gel. After quantification of each sample, 500 ng of each genomic DNA sample was submitted to bisulfite conversion using the EZ DNA Methylation Kit (Zymo Research) according to manufacturer’s protocol.

For *cohort 1*, DNA methylation levels were measured using the Illumina Human Methylation 450 K BeadChip (Illumina, Inc., San Diego, CA). DNA methylation level was then estimated at 485,577 CpG sites and the HAPLIN R package was used to extract the raw probe intensity values. These raw values were preprocessed using ENmix R package(Xu, Niu et al. 2021). The following criteria were used to identify low-quality samples:

1. average intensity value across internal control probes less than 5500
2. more than 5% of CpG probes having low-quality data (Illumina detection P value > 10^− 6^, read from less than 3 beads, or outlier value for the probe in the dataset)
3. clear outliers based on visual inspection of a density plot of total intensity.

Next, the low quality CpG probes were defined as follows:

1. more than 5% low-quality data
2. common SNP within the probe’s sequence (minor allele frequency *≥* 0:05 in Europeans based on 1000 Genomes Project data), or probes mapping to multiple genomic locations, or CpGs on X or Y chromosomes
3. CpGs with multiple mode distributions identified with ENmix. More details about the filtering steps can be found in the cited work (Romanowska, Haaland et al. 2020).

For *cohort 2*, after bisulfite conversion, genomic DNA samples were amplified by fluorescence-based, real time quantitative PCR (Methylight)(Eads, Danenberg et al. 2000). Quantitative MethyLight assays (e.g., EpiTect MethyLight Assays) consist of two probes, one methylation specific and the other nonmethylation specific, which can be used in a single real-time PCR reaction. This enables highly accurate quantitative methylation analysis, due to the simultaneous detection of methylated and unmethylated DNA. We used locus-specific PCR primers flanking an oligonucleotide probe with a 5’ fluorescent reporter dye to detect methylated (SUN) and unmethylated (FAM) alleles and a 3’ quencher dye (3IABkFQ). The primers and fluorescent probes were designed against bisulfite-converted DNA sequence and quantitative information was obtained in real time. Serial dilutions of the EpiTect Control DNAs (Qiagen) were included on each plate to generate a standard curve and to verify plate to plate consistency. The PCR amplification was performed in a 384-well plate format and each sample was run in duplicate. EpiTect MethyLight assays enable the direct quantification of the methylation degree in a sample by taking the threshold cycles (C_T_) determined in the SUN channel with the probe detecting methylated DNA or in the FAM channel with the probe detecting unmethylated DNA. Ten nanograms of bisulfite converted, methylated and unmethylated human control DNA, or defined mixtures of both DNAs were used for methylation quantification. The methylation degree of each sample was calculated from the average of C_T_ values in SUN (C_T(CG)_) and FAM (C_T(TG)_) channel, obtained in quantitative real-time PCR using the formula described in (Cottrell, Jung et al. 2007): C_meth_ = 100/[1 + 2^(C^_T(CG)_^− C^_T(TG)_^)^]%; where C_T(CG)_ is the methylated signal obtained by the threshold cycle of the CG reporter (SUN channel), and C_T(TG)_ is the unmethylated signal obtained by the threshold cycle of the CG reporter (FAM channel).

#### Statistical analysis

Discovery Analysis (Cohort 1): We performed meQTL analysis using the R package MatrixEQTL (Shabalin 2012), testing associations between each of the 10 SNPs and all 407,513 high-quality CpG sites that passed quality control, totaling approximately 4.08 million tests. Linear regression models assessed the relationship between CpG methylation levels (dependent variable) and SNP genotype (independent variable, additive model: 0, 1, or 2 copies of the minor allele), with cleft status included as a covariate. The statistical model was *Methylation ~ Genotype + Cleft_Status + Covariates*. We applied Benjamini-Hochberg false discovery rate (FDR) correction for multiple testing across the ~ 4.08 million tests performed (10 SNPs × 407,513 CpGs). The discovery threshold of *P* < 0.002 and FDR < 0.4 was chosen based on principles of two-stage study designs (Satagopan, Verbel et al. 2002; van den Oord and Sullivan 2003; Xie, Whitehurst et al. 2018), where liberal discovery thresholds maximize sensitivity for subsequent independent validation. This approach accepts a higher proportion of false positives at the discovery stage, which are then filtered through validation in an independent cohort.

##### Validation Analysis (Cohort 2)

We tested the 23 candidate SNP-CpG associations identified in Cohort 1 using the 358 discordant sibling pairs and a linear regression model implemented in MatrixEQTL. Given that only 23 specific hypotheses were tested in validation, we report both nominal P-values and FDR-corrected q-values. Associations achieving *P* < 0.05 with concordant direction of effect were considered validated at the nominal level. To assess the robustness of our findings to multiple testing correction, we also applied Bonferroni correction for 23 tests (significance threshold *P* < 0.0022).

##### Mediation Analysis

To evaluate whether DNA methylation mediates the association between SNP variants and OFC status, we performed formal causal mediation analysis for all 9 validated SNP-CpG pairs using the counterfactual framework implemented in the *mediation* R package (Tingley et al. 2014). For each SNP-CpG pair, we first constructed a mediator model using linear regression to assess the effect of the SNP on DNA methylation levels. Next, an outcome model was fitted using logistic regression to estimate the joint effects of the SNP and DNA methylation on OFC status (affected vs. unaffected). The Average Causal Mediation Effect (ACME), Average Direct Effect (ADE), and total effect were estimated with 1,000 bootstrap simulations to obtain 95% confidence intervals.

Genotype × Tissue Interaction Testing: To formally assess tissue-specific meQTL effects, we employed linear regression models including an interaction term (genotype × tissue type) for each validated SNP-CpG association. The model was specified as: *Methylation ~ Genotype + Tissue + Genotype × Tissue + Covariates*. A significant interaction term (*P* < 0.05) was interpreted as evidence for genuine tissue-specificity of the meQTL effect beyond power differences between tissues. We also calculated the correlation between meQTL effect sizes in blood versus saliva across all validated associations.

Differential Methylation Analysis: To assess methylation differences independent of SNP effects, we performed paired Student’s t-tests comparing methylation levels between affected and unaffected siblings within each discordant pair, stratified by tissue type (blood, saliva, and combined).

##### Functional Annotation

 We investigated the regulatory potential of validated meQTL-associated CpG sites using the GeneHancer database(Fishilevich, Nudel et al. 2017),—a regulatory element database containing genomic coordinates of known enhancer and promoter elements, including those active during craniofacial development (Wilderman, VanOudenhove et al. 2018; Yankee, Oh et al. 2023). We compared these coordinates to our CpG sites to: (1) investigate whether validated CpG sites overlapped with regulatory elements and (2) prioritize the most likely CpG-target genes in relevant tissues (e.g., epithelial and mesenchymal tissues) based on GeneHancer in silico prediction tools.

To characterize the developmental timing and population-level validity of identified meQTLs, we queried the mQTL Database (http://www.mqtldb.org/) (Gaunt, Shihab et al. 2016). This resource contains genome-wide cis and trans meQTL data from approximately 1000 mother-child pairs with methylation measurements at five life stages: birth, childhood (age 7), adolescence (age 15–17), middle age (mean age 48), and pregnancy. We systematically searched for each of our 10 SNPs to determine:

1. Whether they function as meQTLs in this independent population
2. Which CpG sites are affected
3. Whether effects are cis or trans-acting
4. The developmental timing of peak effects. This cross-referencing provides external validation and developmental context for our disease-specific findings.

## Results

### Discovery of meQTL associations in the case-control cohort

Analysis of 10 well-established OFC-associated SNPs against 407,513 high-quality CpG sites in the discovery cohort identified 23 significant SNP-CpG associations meeting our criteria (*P* < 0.002, FDR < 0.4) (Table 2) in the discovery analysis. The strongest association was observed between rs4752028 (*VAX1*) and cg08319991 (*UCHL1*) with *P* = 2.83 × 10^− 8^, followed closely by rs8001641 (*SPRY2*) and cg19191560 (*LGR4*) with *P* = 2.88 × 10^− 8^. The third most significant association involved rs7590268 (*THADA*) and cg06873343 (*TTYH3*) at *P* = 7.72 × 10^− 8^.

The 23 identified meQTL associations displayed diverse regulatory architectures. Seventeen associations represented cis-acting effects, where the SNP and associated CpG site were located within 1 Mb on the same chromosome. Six associations represented trans-acting effects, with SNP-CpG pairs located on different chromosomes or separated by > 1 Mb. For cis-acting associations, the median distance between SNP and CpG was 45 kb (range: 2–890 kb). The CpG sites mapped to diverse genomic contexts: promoter regions (*n* = 5), enhancer regions (*n* = 8), gene bodies (*n* = 6), and intergenic regions (*n* = 4). Among the 9 validated associations, 6 were cis-acting and 3 were trans-acting, indicating that both local and distal regulatory mechanisms contribute to OFC susceptibility through DNA methylation.

Several associations involved the same SNPs affecting multiple CpG sites, suggesting pleiotropic regulatory effects. The rs4752028 (*VAX1*) variant was associated with both cg08319991 (*UCHL1*, *P* = 2.83 × 10^− 8^) and cg11876012 (*AFAP1L2*, *P* = 1.41 × 10^− 7^), indicating that this variant may influence multiple downstream targets. Similarly, rs227731 (*NOG*) showed associations with multiple CpG sites including cg08592707 (*SKA2*, *P* = 1.59 × 10^− 5^) and cg01964121 (*PPM1E*, *P* = 8.11 × 10^− 4^). The well-known 8q24 variant rs987525, representing one of the most validated OFC risk loci across populations, showed significant association with cg16561172 (*MYC*) at *P* = 7.04 × 10^− 4^.

The identified meQTL associations included both cis-acting effects, where SNPs and CpG sites were located on the same chromosome or in proximity, such as rs1873147 (*TPM1*) with cg11936410 (*TPM1*) and rs560426 (*ABCA4/ARHGAP29*) with cg00405232 (*ABCA4*), as well as trans-acting effects across chromosomes, indicating long-range regulatory mechanisms that may involve chromatin looping or other three-dimensional nuclear architecture.

**Table 2:** Most significant meQTLs resulting from case-control study

SNP	SNP gene	CpG	CpG target gene	*P* value	Action
rs4752028	*VAX1*	cg08319991	*UCHL1*	2.83 × 10^− 8^	Trans
*rs8001641*	*SPRY2*	cg19191560	*LGR4*	2.88 × 10^− 8^	Trans
*rs7590268*	*THADA*	cg06873343	*N/A*	7.72 × 10^− 8^	Trans
rs7078160	*VAX1*	cg08319991	*UCHL1*	1.11 × 10^− 7^	Trans
*rs3758249*	*FOXE1*	cg20308679	*FRZB*	1.29 × 10^− 7^	Trans
*rs4752028*	*VAX1*	cg11876012	*AFAP1L2*	1.41 × 10^− 7^	Trans
*rs227731*	*NOG*	cg08592707	*PPM1E*	1.59 × 10^− 5^	Cis
rs1873147	*TPM1*	cg11936410	*TPM1*	5.41 × 10^− 4^	Cis
rs560426	*ABCA4/ARHGAP29*	cg25196715	*ABCA4*	6.72 × 10^− 4^	Cis
*rs987525*	*8q24*	cg16561172	*MYC*	7.04 × 10^− 4^	Cis
rs560426	*ABCA4/ARHGAP29*	cg00405232	*ABCA4*	7.93 × 10^− 4^	Cis
*rs227731*	*NOG*	cg01964121	*SKA2*	8.11 × 10^− 4^	Cis
*rs227731*	*NOG*	cg10303698	*CUEDC1*	8.74 × 10^− 4^	Cis
rs1873147	*TPM1*	cg24483493	*HERC1*	9.14 × 10^− 4^	Cis
rs4752028	*VAX1*	cg08329473	*AFAP1L2*	9.29 × 10^− 4^	Cis
rs13041247	*MAFB*	cg18347630	*CHD6*	1.02 × 10^− 3^	Cis
*rs7078160*	*VAX1*	cg08329473	*AFAP1L2*	1.06 × 10^− 3^	Cis
rs1873147	*TPM1*	cg16659880	*TPM1*	1.11 × 10^− 3^	Cis
rs7078160	*VAX1*	cg09487139	*LINC02626*	1.38 × 10^− 3^	Cis
rs13041247	*MAFB*	cg00514723	*LPIN3*	1.39 × 10^− 3^	Cis
rs742071	*PAX7*	cg20940024	*N/A*	1.56 × 10^− 3^	Cis
rs1873147	*TPM1*	cg19631779	*TPM1*	1.67 × 10^− 3^	Cis
rs13041247	*MAFB*	cg17103269	*PLCG1*	1.78 × 10^− 3^	Cis

SNPs in italic indicate SNPs that have an meQTL role in other tissues or phenotypes

### Validation of meQTL associations in cleft-discordant sibling pairs

Of the 23 candidate meQTL associations identified in the discovery phase, 9 were successfully validated in the independent cohort of same-sex cleft-discordant sibling pairs (Table 3). The validation analysis revealed distinct tissue-specific patterns and combined effects across blood and saliva samples.

In blood samples, three meQTL associations achieved statistical significance. The association between rs13041247 (*MAFB*) and cg18347630 (*PLCG1*) reached significance at *P* = 3.93 × 10^− 2^, while rs7590268 (*THADA*) showed association with cg06873343 (*TTYH3*) at *P* = 3.91 × 10^− 2^. Additionally,

**Table 3:** meQTLs validated with cohort 2 (discordant sibling pairs)

sample	affected	unaffected	snps	snp gene	gene	cpg gene	pvalue
All	305	305	rs987525	*8q24*	cg16561172	*MYC*	2.78 × 10^− 5^
Blood	138	138	rs987525		cg16561172		6.12 × 10^− 1^
Saliva	167	167	rs987525		cg16561172		9.63 × 10^− 6^
All	300	300	rs227731	*NOG*	cg10303698	*CUEDC1*	1.50 × 10^− 3^
Blood	110	110	rs227731		cg10303698		5.08 × 10^− 1^
Saliva	190	190	rs227731		cg10303698		5.95 × 10^− 2^
All	354	354	rs227731		cg08592707	*PPM1E*	1.54 × 10^− 2^
Blood	163	163	rs227731		cg08592707		3.66 × 10^− 1^
Saliva	191	191	rs227731		cg08592707		5.34 × 10^− 1^
All	297	297	rs560426	*ABCA4/ARHGAP29*	cg25196715	*ABCA4*	3.16 × 10^− 2^
Blood	126	126	rs560426		cg25196715		3.88 × 10^− 1^
Saliva	171	171	rs560426		cg25196715		3.81 × 10^− 2^
All	356	356	rs7590268	*THADA*	cg06873343	*N/A*	3.08 × 10^− 1^
Blood	165	165	rs7590268		cg06873343		3.91 × 10^− 2^
Saliva	191	191	rs7590268		cg06873343		9.14 × 10^− 1^
All	353	353	rs13041247	*MAFB*	cg18347630	*CHD6*	2.13 × 10^− 1^
Blood	160	160	rs13041247		cg18347630		3.93 × 10^− 2^
Saliva	193	193	rs13041247		cg18347630		3.38 × 10^− 1^
All	333	333	rs7078160	*VAX1*	cg09487139	*LINC02626*	7.30 × 10^− 1^
Blood	157	157	rs7078160		cg09487139		4.60 × 10^− 2^
Saliva	176	176	rs7078160		cg09487139		2.75 × 10^− 1^
All	340	340	rs8001641	*SPRY2*	cg19191560	*LGR4*	4.70 × 10^− 2^
Blood	158	158	rs8001641		cg19191560		9.33 × 10^− 2^
Saliva	182	182	rs8001641		cg19191560		3.72 × 10^− 2^
All	318	318	rs3758249	*FOXE1*	cg20308679	*FRZB*	1.10 × 10^− 1^
Blood	131	131	rs3758249		cg20308679		2.92 × 10^− 1^
Saliva	187	187	rs3758249		cg20308679		3.80 × 10^− 2^

rs7078160 (*VAX1*) demonstrated association with cg09487139 at *P* = 4.60 × 10^− 2^; this CpG site maps to a human gene located on chromosome 10 that produces a long non-coding RNA (lncRNA) molecule.

Analysis of saliva samples revealed distinct tissue-specific patterns. The rs3758249 (*FOXE1*) variant showed significant association with cg20308679 (*FRZB*) at *P* = 3.80 × 10^− 2^, representing an important connection between two genes previously linked to OFC development. The rs8001641 (*SPRY2*) variant was associated with cg19191560 (*LGR4*) at 3.72 × 10^− 2^. Most remarkably, rs987525 (8q24) demonstrated a highly significant association with cg16561172 (*MYC*) at *P* = 9.63 × 10^− 6^, representing the strongest statistical signal in our entire validation analysis. The rs560426 (*ABCA4/ARHGAP29*) variant showed association with cg25196715 (*ABCA4/ARHGAP29*) at 3.81 × 10^− 2^, demonstrating a cis-acting regulatory effect.

When blood and saliva samples were analyzed together, several associations achieved statistical significance. The rs227731 (*NOG*) variant showed associations with two distinct CpG sites, cg08592707 (*PPM1E*) with *P* = 1.54 × 10^− 2^ and cg10303698 (*CUEDC1*) with *P* = 1.50 × 10^− 3^, with the latter representing one of the most significant findings in the validation cohort. The rs8001641 (*SPRY2*) association with cg19191560 (*LGR4*) remained significant at *P* = 4.70 × 10^− 2^ in the combined analysis. The 8q24 association remained highly robust, with rs987525 and cg16561172 (*MYC*) showing *P* = 2.78 × 10^− 5^ in the combined analysis. The rs560426 (*ABCA4/ARHGAP29*) association with cg25196715 (*ABCA4/ARHGAP29*) achieved *P* = 3.16 × 10^− 2^ in the combined analysis.

The most statistically robust finding across all analyses was the rs987525 (8q24) association with cg16561172 (*MYC*), which achieved genome-wide significance in saliva samples and remained highly significant in the combined analysis, providing compelling evidence for a functional mechanism underlying this well-established but poorly understood OFC risk locus.

### Mediation analysis

To test whether DNA methylation mediates the relationship between genetic variants and OFC risk, we performed formal causal mediation analysis for the 9 SNP-CpG pairs validated in cohort 2 (Table 4). Across all tested pairs, the Average Causal Mediation Effects (ACME) were not statistically significant (all *P* > 0.05), with 95% confidence intervals overlapping zero. For the rs987525–cg16561172 (*MYC*) association, the ACME estimate was 0.003 (95% CI −0.012 to 0.014, *P* = 5.7 × 10^− 1^). Similarly, for the rs227731–cg08592707 (*PPM1E*) association, the ACME was − 0.002 (95% CI −0.008 to 0.001, *P* = 3.46 × 10^− 1^).

The absence of significant single-CpG mediation suggests that for these loci, individual CpG sites may not capture the full complexity of the genotype-phenotype association. The genetic influence on OFC risk may instead involve coordinated epigenetic mechanisms whereby multiple methylation sites or broader chromatin changes collectively mediate risk, rather than the single-site effects tested herein.

**Table 4:** formal causal mediation analysis for the 9 SNP-CpG pairs validated in cohort 2

rsid	cpgid	Effect	Estimate	CI_Lower	CI_Upper	P_Value
rs7590268	cg06873343	ACME (Indirect)	−0.0040	−0.0127	0.0019	1.98 × 10^− 1^
rs7590268	cg06873343	ADE (Direct)	0.0406	−0.0261	0.1065	2.16 × 10^− 1^
rs227731	cg08592707	ACME (Indirect)	−0.0015	−0.0080	0.0011	3.46 × 10^− 1^
rs227731	cg08592707	ADE (Direct)	0.0176	−0.0323	0.0715	4.88 × 10^− 1^
rs7078160	cg09487139	ACME (Indirect)	−0.0004	−0.0046	0.0018	7.16 × 10^− 1^
rs7078160	cg09487139	ADE (Direct)	0.0168	−0.0464	0.0757	6.12 × 10^− 1^
rs227731	cg10303698	ACME (Indirect)	−0.0002	−0.0058	0.0044	9.20 × 10^− 1^
rs227731	cg10303698	ADE (Direct)	0.0175	−0.0395	0.0785	5.36 × 10^− 1^
rs987525	cg16561172	ACME (Indirect)	0.0031	−0.0116	0.0139	5.72 × 10^− 1^
rs987525	cg16561172	ADE (Direct)	0.0458	−0.0098	0.1100	1.10 × 10^− 1^
rs13041247	cg18347630	ACME (Indirect)	0.0018	−0.0021	0.0075	4.10 × 10^− 1^
rs13041247	cg18347630	ADE (Direct)	−0.0518	−0.1058	0.0037	6.60 × 10^− 2^
rs8001641	cg19191560	ACME (Indirect)	−0.0035	−0.0118	0.0018	2.30 × 10^− 1^
rs8001641	cg19191560	ADE (Direct)	0.0051	−0.0464	0.0599	8.70 × 10^− 1^
rs3758249	cg20308679	ACME (Indirect)	−0.0015	−0.0091	0.0044	6.24 × 10^− 1^
rs3758249	cg20308679	ADE (Direct)	0.0257	−0.0277	0.0834	4.26 × 10^− 1^
rs560426	cg25196715	ACME (Indirect)	0.0034	−0.0016	0.0110	2.52 × 10^− 1^
rs560426	cg25196715	ADE (Direct)	−0.0181	−0.0801	0.0360	5.56 × 10^− 1^

*ACME* average causal mediation effects, *ADE* average direct effect, *CI* confidence interval

### Tissue-specificity of meQTL effects

To distinguish true tissue-specific regulation from power differences between blood and saliva analyses, we performed formal genotype × tissue interaction testing (Table 5). Effect sizes showed strong positive correlation between tissues (*r* = 0.81), indicating that the direction and magnitude of genetic regulation are largely conserved.

However, two associations demonstrated significant tissue-specific effects. The rs987525–cg16561172 (*MYC*) association showed a significant genotype × tissue interaction (estimate = 0.100, *P* = 1.00 × 10^− 3^), with substantially stronger effects in saliva compared to blood. This tissue-specificity is particularly relevant to OFC pathogenesis given the ectodermal origin of palatal epithelium and the presence of epithelial cells in saliva samples. The rs7078160–cg09487139 association also showed significant tissue-specificity (estimate = − 0.030, *P* = 2.9 × 10^− 2^), with stronger effects in blood.

For the remaining 7 validated associations, genotype × tissue interaction terms were not significant (*P* > 0.05), suggesting that observed differences in tissue-specific significance levels reflect variations in statistical power rather than true biological divergence in meQTL effects.

**Table 5:** formal genotype × tissue interaction testing with the 9 SNP-CpG pairs validated in cohort 2

rsid	cpgid	estimate	std.error	statistic	*p*.value	term
rs987525	cg16561172	0.1	0.03	3.3	**1.00 × 10** ^**− 3**^	snp: tissueSaliva
rs7078160	cg09487139	−0.03	0.01	−2.2	**2.90 × 10** ^**− 2**^	snp: tissueSaliva
rs7590268	cg06873343	0.03	0.02	1.61	1.08 × 10^− 1^	snp: tissueSaliva
rs560426	cg25196715	0.05	0.07	0.73	4.65 × 10^− 1^	snp: tissueSaliva
rs8001641	cg19191560	0	0	−0.71	4.77 × 10^− 1^	snp: tissueSaliva
rs227731	cg08592707	−0.03	0.05	−0.55	5.85 × 10^− 1^	snp: tissueSaliva
rs3758249	cg20308679	−0.04	0.09	−0.49	6.24 × 10^− 1^	snp: tissueSaliva
rs227731	cg10303698	−0.05	0.14	−0.38	7.03 × 10^− 1^	snp: tissueSaliva
rs13041247	cg18347630	0	0.01	0.09	9.26 × 10^− 1^	snp: tissueSaliva

### Differential DNA methylation analysis between discordant siblings

Independent of SNP effects, paired t-test analyses comparing methylation levels between affected and unaffected siblings revealed significant differential methylation at specific CpG sites (Table 6). The cg06873343 (*TTYH3*) site showed the strongest and most consistent differential methylation signal across all analyses. In the combined analysis of 358 sibling pairs, this site achieved *P* = 4.17 × 10^− 3^, with consistent significance maintained in blood-specific analysis of 165 pairs (*P* = 3.76 × 10^− 2^) and saliva-specific analysis of 193 pairs (*P* = 4.94 × 10^− 2^). This remarkable consistency across different tissue types strongly indicates robust differential methylation at this locus that is independent of tissue-specific factors.

The cg17103269 (*LPIN3*) site demonstrated clear blood-specific differential methylation with high statistical significance in blood samples from 166 pairs (*P* = 1.68 × 10^− 3^), representing one of the strongest signals in our differential methylation analysis. However, this effect was not observed in saliva.

**Table 6:** Differential methylation levels between sibling pairs and subdivided by groups

Paired T-Test					
CpG site	Chr position	Closest gene	# of sib pairs	Group	*P* value
cg06873343	chr7:2,667,608	*TTYH3*	358	All	**4.17 × 10** ^**− 3**^
			165	Blood	**3.76 × 10**^**− 2**^
			193	Saliva	**4.94 × 10** ^**− 2**^
cg17103269	chr20:39,972,305	*LPIN3*	360	All	2.54 × 10^− 1^
			166	Blood	**1.68 × 10** ^**− 3**^
			194	Saliva	8.75 × 10^− 1^
cg19191560	chr11:27,492,758	*LGR4*	340	All	**5.28 × 10** ^**− 2**^
			158	Blood	**5.87 × 10** ^**− 2**^
			182	Saliva	2.59 × 10^− 1^
cg25196715	chr1:94,489,792	*ABCA4/ARHGAP29*	297	All	1.27 × 10−1
			126	Blood	**7.01 × 10** ^**− 2**^
			171	Saliva	5.61 × 10^− 1^

*P-*values in bold are borderline significant (*p* < 0.09)

samples from 194 pairs (*P* = 8.75 × 10^− 1^) or in the combined analysis of 360 pairs (*P* = 2.54 × 10^− 1^), suggesting tissue-specific regulatory mechanisms.

The cg19191560 (*LGR4*) site showed borderline significance in the combined analysis of 340 pairs (*P* = 5.28 × 10^− 2^), with similar trends observed in blood-specific analysis of 158 pairs (*P* = 0.059) but not in saliva-specific analysis of 182 pairs (*P* = 2.59 × 10^− 1^). The cg25196715 (*ABCA4/ARHGAP29*) site showed suggestive evidence for differential methylation, particularly in blood samples from 126 pairs (*P* = 7.01 × 10^− 2^), though this did not reach conventional statistical significance thresholds.

### Functional annotation of validated meQTL sites

Analysis using the GeneHancer and craniofacial enhancers databases (Wilderman, VanOudenhove et al. 2018; Yankee, Oh et al. 2023) revealed that several validated CpG sites overlapped with known regulatory elements active during craniofacial development. The cg16561172 site associated with rs987525 (8q24) mapped to a mesendoderm-active enhancer region (ENSR8_CGN7Z) located upstream of *MYC*, providing the first mechanistic insight into how this long-standing mystery locus may influence OFC risk through epigenetic regulation of this critical developmental gene. The cg08592707 site linked to rs227731 (*NOG*) resided within a promoter region (ENSR17_9TBN3) active in dermal fibroblasts, mesenchymal stem cells, and mesendoderm, which is consistent with its association with *PPM1E*, a gene known to be involved in osteoblast proliferation and bone formation processes critical for proper craniofacial development.

The cg20308679 site associated with rs3758249 (*FOXE1*) mapped to a promoter region (ENSR2_94NKPT) active in dermal fibroblasts, foreskin fibroblasts, mesenchymal stem cells, and mesendoderm. This regulatory element annotation supports the biological relevance of the *FOXE1-FRZB* association, as both genes have established roles in craniofacial development and the Wnt signaling pathway.

Cross-referencing our findings with the mQTL Database confirmed that seven of our identified SNPs function as meQTLs in other tissues and developmental contexts, supporting the validity of our findings while highlighting the tissue-specific and context-dependent nature of some associations (Table 7). This functional annotation analysis supports the biological plausibility of our identified meQTL associations and provides evidence that they represent genuine regulatory interactions rather than spurious statistical associations, establishing a foundation for future functional validation studies.

**Table 7:** Functional analysis of SNPs functioning as meQTLs in other tissues and developmental contexts

SNP	Gene/Locus	mQTL database evidence	Developmental pattern	Regulatory mechanism
rs7078160	*VAX1*	30 associations across 5 life stages. Strongest association with cg11398452 (*VAX1*), *P* = 1.01 × 10^− 29^ (childhood)	Childhood peak: 10 associations, strongest effects. Persistent: Birth → Pregnancy. Mainly cis-acting: 29/30 associations	Primarily targets *VAX1* regulatory region Consistent methylation control across development.
rs4752028	*VAX1*	29 associations across all 5 life stages. Strongest: cg11398452 (*VAX1*), *P* = 9.41 × 10^− 30^ (childhood)	Childhood dominant: 10 associations. Stable effects: Birth through pregnancy. Mainly cis-acting: 28/29 associations	Co-regulates with rs7078160. Fine-tunes *VAX1* methylation landscape.
rs3758249	*FOXE1*	10 associations across all 5 life stages. Strongest: cg13791254, *P* = 4.99 × 10^− 30^ (childhood)	Early childhood peak: Strongest effects Sustained: Adolescence through pregnancy. Mainly cis: 9/10 associations	Targets *FOXE1* regulatory elements. Critical for thyroid/craniofacial development.
rs7590268	*THADA*	10 associations across all 5 life stages. Strongest: cg04684014 (*THADA*), *P* = 2.23 × 10^− 36^ (childhood)	Childhood maximum. Pregnancy important. Strong effects. Entirely cis-acting: 10/10 associations	Regulates *THADA* methylation. Metabolic-developmental interface.
rs8001641	*SPRY2*	5 associations across 4 life stages Strongest: cg20100768, *P* = 3.35 × 10^− 19^ (childhood)	Childhood strongest. Not active in pregnancy. Mostly cis: 4/5 associations	Trans-chromosomal effects to Chr13 *SPRY2*-mediated growth control.
rs987525	8q24	1 association in middle age only. Target: cg03645865, *P* = 1.65 × 10^− 8^	Middle age specific. Only active timepoint. Trans-acting: Chr8 → Chr7. Adult-onset effects	Long-range trans regulation. Age-dependent activation pattern.

## Discussion

This study represents the first comprehensive investigation of methylation quantitative trait loci (meQTLs) for orofacial clefts, addressing a critical gap in understanding how genome-wide association study (GWAS) variants influence disease risk through epigenetic mechanisms (Villicaña and Bell 2021; Villicaña, Castillo-Fernandez et al. 2023). Our approach successfully identified and validated nine meQTL associations between established OFC risk variants and DNA methylation sites, providing mechanistic insights that bridge genetic variation with functional consequences during craniofacial development.

The most significant finding of our study concerns the long-standing mystery surrounding the 8q24 locus and its association with OFC risk. The rs987525 variant at 8q24 has been consistently identified across multiple GWAS as one of the strongest genetic risk factors for orofacial clefts (Birnbaum, Ludwig et al. 2009; Grant, Wang et al. 2009; Beaty, Murray et al. 2010; Ludwig, Mangold et al. 2012; Salagovic, Klimcakova et al. 2017; Butali, Mossey et al. 2019) yet the functional mechanism underlying this association has remained elusive for over a decade. Our meQTL analysis provides compelling mechanistic explanation for this association by evidencing that rs987525 significantly modulates methylation at cg16561172, located upstream of the *MYC* gene (*P* = 9.6 × 10^− 6^). This methylation site overlaps with a mesendoderm-active enhancer region (Fishilevich, Nudel et al. 2017; Wilderman, VanOudenhove et al. 2018; Yankee, Oh et al. 2023), suggesting that the 8q24 risk allele disrupts normal epigenetic regulation of *MYC* during critical developmental windows.

The *MYC* gene encodes a master regulator of cellular proliferation, growth, and differentiation (Meyer and Penn 2008; Dang 2012), processes that are fundamentally critical during craniofacial morphogenesis (Trainor 2014; Twigg and Wilkie 2015). Even subtle dysregulation of *MYC* expression could lead to defects in palatal shelf growth, fusion timing, or both, explaining how this distant regulatory variant influences OFC susceptibility through long-range epigenetic control (Karsenty and Wagner 2002; Long and Ornitz 2013).

The unique temporal pattern of rs987525 in the mQTL Database (Gaunt, Shihab et al. 2016)showing methylation effects exclusively during middle age through trans-chromosomal regulation, suggests this variant may have pleiotropic effects across the lifespan. This age-specific activation pattern distinguishes the 8q24 locus from other OFC variants and may explain why this region has been difficult to characterize functionally using traditional developmental biology approaches that focus on embryonic stages (Santagati and Rijli 2003; Chai and Maxson 2006). The finding that this variant exerts its strongest effects through adult methylation patterns indicates that the 8q24 locus may influence OFC risk through maternal epigenetic factors or transgenerational inheritance mechanisms (Gapp, Jawaid et al. 2014; Heard and Martienssen 2014), opening new avenues for understanding gene-environment interactions in cleft etiology (Waterland and Michels 2007; Heijmans, Tobi et al. 2008).

To rigorously assess whether observed differences between blood and saliva reflected true biological divergence or merely power differences, we performed formal genotype × tissue interaction testing for all validated meQTLs. Effect sizes showed strong positive correlation between tissues (*r* = 0.81), indicating that the direction and magnitude of genetic effects on methylation are largely conserved across tissue types. However, two associations demonstrated significant tissue-specific regulation: rs987525–cg16561172 (*MYC*; interaction *P* = 1.00 × 10^− 3^) and rs7078160–cg09487139 (interaction *P* = 2.9 × 10^− 2^). For the remaining associations, the lack of significant interaction terms suggests that differential significance across tissues was driven by variations in statistical power rather than true biological divergence.

The tissue-specificity of the 8q24–*MYC* meQTL has important implications for future functional studies. Blood-based methylation analyses, which dominate the field due to sample accessibility, may underestimate or miss entirely the regulatory effects of this variant. Our findings suggest that oral epithelial tissues or saliva-derived samples may be more appropriate for studying the functional consequences of the 8q24 locus in OFC etiology.

To evaluate whether DNA methylation mediates the relationship between genetic variants and OFC risk, we performed formal causal mediation analysis using the counterfactual framework (Tingley, Yamamoto et al. 2014). Across all tested SNP-CpG pairs, the Average Causal Mediation Effects (ACME) were not statistically significant, with 95% confidence intervals overlapping zero. Due to the limits of statistical analyses investigating CpG sites in isolation, this finding warrants careful interpretation.

The absence of significant single-CpG mediation does not negate the biological relevance of our meQTL findings. Rather, it suggests that the epigenetic contribution to OFC risk likely involves coordinated regulation across multiple methylation sites or broader chromatin landscape changes, rather than effects at individual CpG sites. This interpretation is consistent with emerging understanding that genetic effects on complex traits often operate through distributed epigenetic mechanisms (Relton and Davey Smith 2012; Villicaña, Castillo-Fernandez et al. 2023). Large-scale studies have demonstrated that meQTLs frequently influence multiple CpG sites within regulatory regions, and that the aggregate effect of these coordinated changes may be required to meaningfully alter gene expression and downstream phenotypes.

The robust meQTL associations we identified nonetheless represent a critical first step in elucidating the causal pathway from genotype to phenotype. Our findings pinpoint specific regulatory regions—particularly the 8q24–*MYC* enhancer—where genetic variation influences the epigenetic landscape. Future studies incorporating regional methylation patterns, multi-CpG scores, or experimental manipulation of methylation at these loci will be needed to fully characterize the mediating mechanisms.

Our cross-referencing with longitudinal methylation data from the mQTL Database (Gaunt, Shihab et al. 2016) revealed that OFC-associated variants primarily exert their methylation effects during childhood, aligning with the critical periods of craniofacial development (Sperber, Saxena et al. 2008; Marazita 2012). Five of the six validated SNPs (rs7078160, rs4752028, rs3758249, rs7590268, and rs8001641) showed their strongest methylation associations during childhood, with effect sizes reaching genome-wide significance levels. This temporal clustering provides compelling evidence that these genetic variants influence OFC risk by modulating epigenetic patterns during developmentally relevant windows rather than through constitutive effects across all life stages (Burdge and Lillycrop 2010; Waterland, Kellermayer et al. 2010).

Our meQTL analysis revealed associations involving genes not traditionally linked to craniofacial development, expanding the known molecular landscape underlying OFC etiology. The identification of significant associations involving *TTYH3* (chloride channel) (Suzuki and Mizuno 2004; Halleran, Sehdev et al. 2015), *PPM1E* (osteoblast proliferation regulator) (Li, Zhu et al. 2017; Kanazawa, Takeno et al. 2018), *PLCG1* (cell growth and migration) (Tao, Han et al. 2023), and *LGR4* (Wnt signaling modulator) (de Lau, Barker et al. 2011; Glinka, Dolde et al. 2011) suggests that OFC susceptibility extends beyond canonical palatogenesis pathways to encompass broader cellular processes including ion transport, metabolic regulation, and immune homeostasis (Sauka-Spengler and Bronner-Fraser 2008; Theveneau and Mayor 2012).

The *TTYH3* association is particularly noteworthy given its consistent differential methylation across tissue types and its meQTL association with *THADA*. *TTYH3* encodes a large-conductance chloride channel with expression during embryonic nervous system development, suggesting potential roles in neural crest cell migration or epithelial-mesenchymal transitions during facial morphogenesis (Thiery, Acloque et al. 2009; Nieto, Huang et al. 2016). The *PPM1E* association links OFC risk to osteoblast proliferation control, providing a direct connection to bone formation processes critical for proper craniofacial structure development (Karsenty, Kronenberg et al. 2009; Long 2011).

These findings suggest potential therapeutic avenues that might not have been evident through genetic analysis alone. Targeting DNA methylation patterns through epigenetic therapies could theoretically modify OFC risk, particularly during critical developmental windows (Sharma, Kelly et al. 2010; Dawson and Kouzarides 2012). However, such approaches would require careful consideration of timing, tissue specificity, and potential off-target effects given the fundamental roles these pathways play in normal development (Sharma, Kelly et al. 2010; Ahuja, Sharma et al. 2016).

The use of cleft-discordant sibling pairs for validation represents a methodological advance that addresses several limitations of traditional case-control studies. By comparing affected and unaffected siblings, we controlled for shared environmental exposures, and population stratification while maintaining the power to detect disease-relevant epigenetic differences (Bell, Pai et al. 2011; Bell and Spector 2011, 2012; Bell and Saffery 2012).

The integration of multiple analytical approaches—including meQTL mapping, differential methylation analysis, formal mediation testing, genotype × tissue interaction analysis, and functional annotation—provides a comprehensive characterization of the identified associations (Villicaña and Bell 2021; Villicaña, Castillo-Fernandez et al. 2023). The high concordance (86%) between our disease-specific validation and population-level methylation effects in the mQTL Database demonstrates that OFC-associated genetic variants influence methylation through mechanisms conserved across different populations and study designs (Grundberg, Meduri et al. 2013; McRae, Powell et al. 2014).

Several limitations should be acknowledged. First, our formal mediation analysis found no evidence for single-CpG mediation, indicating that individual methylation sites do not fully explain the pathway from genetic variation to OFC risk. This suggests more complex epigenetic architectures that our single-CpG approach could not capture, and future studies using regional or network-based methylation measures may be more informative.

Second, the cross-sectional nature of our methylation measurements does not capture dynamic changes in epigenetic patterns that occur during development (Feinberg 2007; Relton and Davey Smith 2010). This limitation is particularly relevant in OFC, where significant regulatory events occur during specific embryonic windows. Third, although we demonstrated tissue-specificity for key associations, blood and saliva may not fully represent the cell populations most relevant to craniofacial development. However, previous studies have shown correlation between DNA methylation levels in blood and lip tissue (Alvizi, Ke et al. 2017; Sharp, Ho et al. 2017; Alvizi, Brito et al. 2022a), and our finding of stronger effects in saliva for the 8q24 locus suggests that oral-derived samples provide a more relevant proxy than blood for studying OFC-related methylation.

Finally, the functional consequences of the methylation changes that we identified remain to be demonstrated through direct experimental validation. Future studies could employ CRISPR-dCas9-based targeted methylation editing at the 8q24 enhancer region, reporter assays testing enhancer activity, or analysis in craniofacial-relevant cell types to confirm effects on *MYC* expression and developmental processes.

Our findings have potential clinical applications for developing biomarkers for OFC risk assessment, particularly in families with existing genetic risk factors (Widschwendter, Fiegl et al. 2007; Teschendorff, Menon et al. 2010). The tissue-specific effects we observed suggest that saliva-based methylation testing might provide a more informative and non-invasive approach for risk stratification than blood-based testing (Park, Park et al. 2011; Langevin, Koestler et al. 2012). However, clinical applications would require validation in larger, more diverse populations.

In summary, our findings contribute to a more complete understanding of OFC etiology by identifying specific meQTLs that link genetic risk variants to epigenetic regulation at developmentally relevant loci. The 8q24–*MYC* association provides the first mechanistic explanation for this major risk locus, with tissue-specific effects that may explain previous difficulties in functional characterization. While individual CpG sites do not fully mediate genetic risk, our findings identify key regulatory regions where coordinated epigenetic changes likely contribute to OFC susceptibility, providing a foundation for future functional studies and potential therapeutic interventions.
